# Caste-Specific Expression Patterns of Immune Response and Chemosensory Related Genes in the Leaf-Cutting Ant, *Atta vollenweideri*

**DOI:** 10.1371/journal.pone.0081518

**Published:** 2013-11-15

**Authors:** Sarah I. Koch, Katrin Groh, Heiko Vogel, Bill S. Hannson, Christoph J. Kleineidam, Ewald Grosse-Wilde

**Affiliations:** 1 Department of Biology, University of Konstanz, Konstanz, Germany; 2 Department of Evolutionary Neuroethology, Max Planck Institute for Chemical Ecology, Jena, Germany; The University of Queensland, Australia

## Abstract

Leaf-cutting ants are evolutionary derived social insects with elaborated division of labor and tremendous colony sizes with millions of workers. Their social organization is mainly based on olfactory communication using different pheromones and is promoted by a pronounced size-polymorphism of workers that perform different tasks within the colony. The size polymorphism and associated behaviors are correlated to distinct antennal lobe (AL) phenotypes. Two worker phenotypes differ in number of olfactory glomeruli in the AL and the presence or absence of an extremely large glomerulus (macroglomerulus), involved in trail-pheromone reception. The males' AL contains three macroglomeruli which are presumably involved in detection of sex-pheromone components. We investigated the antennal transcriptome data of all major castes (males, queens and workers) and two worker subcastes (large and tiny workers). In order to identify putative odorant receptor genes involved in pheromone detection, we identified differentially expressed odorant receptor genes (OR-genes) using custom microarrays. In total, we found 185 OR-gene fragments that are clearly related to ORs and we identified orthologs for 70 OR-genes. Among them one OR-gene differs in relative expression between the two worker subcastes by a factor of >3 and thus is a very promising candidate gene for the trail-pheromone receptor. Using the relative expression of OR-genes in males versus queens, we identified 2 candidates for sex-pheromone receptor genes in males. In addition, we identified genes from all other chemosensory related gene families (13 chemosensory protein genes, 8 odorant binding protein genes, 2 sensory-neuron membrane protein genes, 7 ionotropic receptor genes, 2 gustatory receptor genes), and we found ant-specific expansions in the chemosensory protein gene family. In addition, a large number of genes involved in immune defense exhibited differential expression across the three different castes, and some genes even between the two worker subcastes.

## Introduction

The family of ants (Formicidae) contains over 12 000 described species, with tremendous global diversity; ants contribute to more than half of the total insect biomass [1]. Social division of labor of these eusocial insects is considered as one of the major driving forces for their ecological impact [2]. Probably the most derived colony organization is found in leaf-cutting ants (genus *Atta*), which harvest leaves and cultivate a fungus in huge underground nests. In each colony, several millions of workers are involved in agriculture, building activities and raising the brood of a single queen [3], and alike in all ant species, odors play a prominent role for communication and social organization of the colony. For example, trail-pheromones are used to inform nestmates about profitable food sources, alarm pheromones mediate conjoint defense, and once a year, sex-pheromones serve to find potential mating partners during the nuptial flights of virgin queens and males [4-6].

Division of labor entails different demands and challenges on the castes (queens, males and workers), e.g. some colony members are restricted to the nest interior (tiny workers), others are foraging (large workers), and virgin queens and males once become airborne. While males die right after mating, successful queens will live for 15 and more years. In particular during nest foundation, queens are not protected by the social immune system of the colony and foraging workers are probably more exposed to pathogens than the tiny workers that are exclusively involved in fungus cultivation [7]. Such pronounced differences in behavioral repertoirs and life-histories are probably supported by distinct expression patterns of many genes, including immune genes (for review focusing on social immune systems see Cremer et al. [8]). 

Differential expression of genes involved in olfaction is expected for several reasons. Leaf-cutting ant workers (*Atta vollenweideri*) show distinct odor-guided behaviors with marked differences in sensitivity and discriminatory ability, e.g. to the trail-pheromones [9]. This may lead to the pronounced division of labor along the foraging trails and to differential recruitment to foraging or battle sites [10,11]. Based on behavioral phenotypes and morphological traits, the worker caste can be further subdivided into subcastes. The subcastes also differ in the neuroanatomy of their first olfactory neuropil, the antennal lobe (AL). While the AL of tiny workers, with a head width of less than one millimeter, contains less than 400 glomeruli, the AL of large workers contains more than 420 glomeruli [12]. Only large workers but not tiny workers possess an extremely large glomerulus (macroglomerulus; MG) in their AL. Comparable to many other insect species, the males' AL in *Atta vollenweideri* contains three MGs [13]. Gomeruli are termination and connection sites for olfactory sensory neurons (OSN) with different odor specificity, and the size of a glomerulus correlates with the number of terminating OSNs [14].

The OSNs detect general odors and pheromones; their dendrites are housed in different types of antennal sensilla. In contrast to the majority of other insect orders, in Hymenoptera each olfactory sensillum contains many OSNs per sensillum (7-23 in honeybees and up to 130 in ants) [15-17]. A complex molecular machinery (perireceptor events) is involved in odor reception that finally leads to activation of chemosensory receptors at the OSN dendrites and to the activation of the olfactory signal transduction cascade [18]. Odorant binding proteins (OBPs) supposedly ferry hydrophobic odor molecules through the aqueous sensillum lymph to the OSN membrane [19-21]. Similar to OBPs, chemosensory proteins (CSPs) are present in the sensilla lymph but so far it is unclear whether they are involved in perireceptor events. Vieira et al. [22] proposed that OBP-genes and CSP-genes belong to a larger gene superfamily coding for general binding proteins with similar function, and that CSP-genes are more conserved than OBP-genes. 

The presumably oldest class of chemosensory receptors involved in olfaction is the class of ionotropic receptors that derived from ionotropic glutamate receptors (iGluRs) and are already present in early Protostomia [23,24]. In *Drosophila*, IRs located in the dendritic membrane of coeloconic OSNs (antennal IRs) are involved in olfaction [23].

Members of another chemosensory receptor class, the gustatory receptors (GRs) are mainly located in non-antennal tissues and only few are present in the antenna of insects. For *D. melanogaster*, GRs are classified either as sugar, bitter or CO_2_ receptors [25,26]. The overall number of GR-genes in Hymenoptera is lower than in e.g. *D. melanogaster* or *Anopheles gambiae* [27-30], but sugar receptor genes have also been identified in some Hymenoptera (for *Apis mellifera* and *Nasonia vitripennis* see 26).

The most prominent receptor class involved in insect olfaction are the odorant receptors (ORs). With few exceptions, each OSN expresses only one type of odorant-specific receptor gene [14]. It is likely that OR-genes evolved from GR-genes, thus belonging to the same highly divergent gene superfamily [22,28]. ORs are seven-transmembrane proteins with inverted topology, which together with a conserved odorant receptor co-receptor (ORCo) assemble into functional heteromers; ORCo also acts as a chaperon for the OR partner [31-35].

OSN axons are sorted at the entrance of the AL; in *D. melanogaster* it has been demonstrated that those expressing the same OR-gene innervate the same glomerulus [14,36]. In ants such direct evidence is missing, but the correlation between the number of expressed OR-genes and the number of glomeruli in the AL indicate similar sorting principles [37,38]. 

Recent efforts have identified OR-, IR- and GR-genes in several ant species [37,39], and sex-specific differences of chemosensory related genes have been described [38]. Although a draft genome of a leaf-cutting ant species (*Atta cephalotes*) is available, chemosensory related genes have not been investigated [40]. 

Based on our knowledge of distinct AL-phenotypes and MGs in large workers and males, we expect that three sex-pheromone receptor genes are highly expressed in males and that the trail-pheromone receptor gene is highly expressed in large workers. Recent Ca-imaging studies showed that the releaser component of the trail-pheromone is represented in the MG of large workers [13]. 

In this study, we investigate the antennal transcriptome data of all major castes (males, queens, and workers) and two worker subcastes (large and tiny workers) of the leaf-cutting ant *A. vollenweideri*. Based on microarray data, we performed caste and subcaste-specific GO analysis and investigated differential expression. Since we expected major differences in the expression of chemosensory related genes, we first identified these genes in *A. vollenweideri* and from the genome data available for *A. cephalotes*. In the second step, we searched for genes that may code for pheromone receptors, using a similar approach as has been used for identification of pheromone receptor genes in Lepidoptera [41,42]. The identified chemosensory related gene families of *A. vollenweideri* are compared to other ant species [38] and to two species from different families of the Hymenoptera (*A. mellifera, N. vitripennis*; [29,30]). 

## Materials and Methods

### Ethic Statement

Animals were collected with all necessary permits from the Argentine Republic (Permit Number: 23275/10), the Parques National Rio Pilcomayo and the Federal Republic of Germany. The examined species is neither endangered nor protected. Laboratory rearing of the individuals is in compliance with the laws of the Federal Republic of Germany.

### Animals

Ants (*A. vollenweideri*) were collected in the palm savanna of Grand Chaco, in the north of Argentina. On two field trips in 2009 and 2010, virgin females (referred to as queens in the following), males and workers were collected from different nests in vicinity of the field station NP Rio Pilcomayo (Formosa), and transported alive to the MPI Chemical Ecology, Germany. Antennae of sexuals and workers from the first collection (2009, C.J. Kleineidam) were pooled for initial transcriptome sequencing, while the second collection (2010, J. Fink and S. Koch) was kept separately for microarray studies (data set 1). The very small workers (tiny workers) never leave the subterranean nest, since their only task is to care for the symbiotic fungus. Therefore, we had to obtain them from a laboratory colony that also has been collected in Grand Chaco in 2002 (M. Bollazzi and O. Geissler; data set 2). The laboratory colony was reared in several interconnected plastic boxes (19 x 19 x 8 cm) at the University of Würzburg, and sub-colonies at the University of Konstanz at 25°C, 50-60% relative humidity and 12h/12h light/dark cycle. The workers were fed with leaf material from Rosacea plants for fungus cultivation. Workers were collected from the feeding site as well as from the fungus garden, and were classified as large workers with head width of more than 1.2 mm and as tiny workers with head widths smaller than 0.8 mm. 

The numbers of collected antennae for each caste (queens, males, workers) and subcaste (large workers and tiny workers) were adjusted to the differences in body size. Each sample consisted of approximately 300 antennae of tiny workers, 100 antennae of large workers and 50 antennae of each of the other castes.

### Extraction of total RNA

For totalRNA sequencing, antennae were cut off and kept over liquid nitrogen for cooling. Subsequently, they were homogenized with stainless steal beads for 10-15 min at 50 Hz in a TissueLyser LT (Qiagen, Hilden Germany) and RNA isolated using either Trizol as extraction reagent for large and tiny workers (Sigma Aldrich, St. Louis, USA, data set 2) or the innuPREP RNA Mini Kit (Analytik Jena, Jena, Germany) for the worker, queen and male samples (data set 1).

For microarray studies, the Trizol protocol (Sigma Aldrich, St. Louis, USA) used for RNA extraction of data set 2 samples was adjusted by replacing chloroform with 1-bromo-3-chloro-propane. Subsequently, residual DNA was removed with the Turbo DNA-free Kit, following the manufacturer instruction (Applied Bioscience, Carlsbad, USA) in order to prepare RNA for microarray experiments. RNA concentration was measured photometrically with a NanoDrop ND-1000 and quality of the RNA was controlled with an Agilent 2100 Bioanalyzer. 

### Sequencing and Assembly

Normalized, non-directional cDNA was produced using totalRNA at Evrogen (Moscow, Russia). This cDNA was taken as a template for Roche 454 FLX sequencing; additionally, Illumina Solexa sequencing of non-normalized cDNA was performed. All sequencing was performed at the MPI for Molecular Genetics in Berlin. Sequence reads were sanitized by removal of adaptor sequences and low-quality reads. Roche 454 data was sanitized further by removing reads with high similarity to sequences originating in fungi, plants, or prokaryotes (cutoff E<10^-20^ in default blastn), since such reads were over-represented due to the normalization procedure. The assembly was performed as a hybrid assembly of both Illumina Solexa and Roche 454 FLX data using CLC Genomics Workbench with default settings. Sequencing data is available at EMBL-EBI under the accession number ERP002375.

### GO analysis

GO terms were automatically assigned to contigs using the Blast2GO algorithm [43]. For all categories of the GO-analysis, the frequency of occurrence of specific terms was calculated on different levels (e.g. level 3 for molecular function and level 2 for biological process) for general quality assessment. 

In order to perform caste and subcaste-specific GO-analysis, presence-absence calls from individual microarrays were tested for statistically significant differences using the chi-square test across castes and subcastes, independently for each term. 

### Identification of genes of interest

Genes of interest were identified from the GO analysis by filtering for specific terms, for example “response to stimulus”. Additionally, custom databases for Blast and HMM profile searches were used to identify putative members of the different gene families (immune genes, CSP-, OBP-, SNMP-, IR-, GR-, OR-genes) as described in Grosse-Wilde et al. [44]. All candidates were subsequently manually revised by further analysis using the tblastx algorithm in combination with the nr (non-redundant) database of NCBI. 

In order to remove potential duplicates, contaminants or splice variants, all contigs related to members of either immune response genes or chemosensory genes were aligned, based on predicted protein sequences with respective gene family members of other ant species (*A. cephalotes, C. floridanus or H. saltator*; http://www.antgenomes.org), and in specific cases with limited information from ant species, data from other insect species was included as indicated. 

Predicted ORFs were determined based on Blast hits and translated into protein sequences using the Geneious software (Version 5.5.5). Due to a draft genome backbone assembly (to *A. cephalotes*) or low sequencing coverage of the trancriptome data, some predicted proteins showed in-frame stop codons or ambiguous sites. In these cases, protein sequences were trimmed, in order to remove for example ambiguous sites. For further analysis, only those protein sequences were selected which after trimming were longer than 50 AA and could still be assigned to the respective chemosensory gene family after blasting. 

Predicted protein sequences of receptor genes either contain transmembrane domains or these domains are missing because of the fragmentary nature of the transcriptome data. First, predicted protein sequences for SNMPs, IRs, GRs, and ORs of *A. vollenweideri* were analyzed in TMHMM [45] in order to identify transmembrane helices. Since some predicted proteins from *A. cephalotes* were very short, likely due to inaccurate gene model prediction, all predicted protein sequences of receptor genes were analyzed the same way. Protein sequences from *A. vollenweideri* and *A. cephalotes* containing >1 transmembrane helix were aligned with the predicted protein sequences from genome data of *H. saltator* and *C. floridanus* (http://www.antgenomes.org) using the MAFFT algorithm [46], with a transmembrane substitution matrix PAM250 [47]. Second, the remaining protein sequences lacking transmembrane helices were aligned with the predicted protein sequences from *H. saltator* and *C. floridanus* using the MAFFT algorithm but with a cytosolic substitution matrix PAM250. Finally, both alignments were manually merged in UGENE [48]. Members of CSP- and OBP-genes families were aligned with the predicted protein sequences from *A. mellifera, N. vitripennis* and *Solenopsis invicta* for OBP phylogeny, using the MAFFT algorithm with a cytosolic substitution matrix PAM250. Protein sequences of both *Atta* species were manually curated in merged alignments where necessary. In few cases, the overlapping part of the protein sequences had very high sequence similarity and we therefore kept only one of these sequences, in order to remove duplicates.

After removal of duplicates, we could relate the remaining protein sequences to gene fragments. For gene families that are conserved (immune genes, CSP-, SNMP-, IR-, GR-genes), these protein sequences were used to unambiguously identify orthologs in other insect species. For some of the chemosensory gene families (OBP-genes and OR-genes) such identification is difficult because of the high sequence variability (regarding ants see comments in [49,38]). In these two gene families, two or more non-overlapping protein sequences might relate to a single gene. Therefore, we selected only overlapping protein sequences for phylogenetic analysis to identify orthologs, and for the remaining sequences, we refer to gene fragments that are related to CSP-, OBP- or OR-genes.

### Phylogenetic analysis

For all chemosensory gene families, we selected only overlapping protein sequences, and columns in multiple sequence alignments with <5% sequence information were removed. The entire alignments were trimmed to the region of the consensus most of the *Atta* protein sequences aligned to (mostly 3' ends). All alignment files used to calculate phylogenies are provided as supplementary data (Datasets S1-6). Phylogenies were inferred using a neighbour-joining analysis in combination with a maximum-likelihood analysis in FastTree [50,51]. Results of the respective calculation are included as supplementary information (Datasets S7-12 in newick format). Dendrograms were plotted and colored using the iTOL software [52]. 

### Gene expression analysis

Custom 2x105 k Agilent microarrays (Agilent Technologies, Palo Alto, CA) were designed based on the antennal transcriptome data of *A. vollenweideri*. For all contigs where automatic annotation identified an ORF, two 60mer oligo probes were designed. For contigs with unknown ORF, two additional 60mer oligo probes for opposite reading direction were designed, thus resulting in 4 oligo probes per contig. Additionally, we added three 60mer oligo probes for each identified and revised OR-contig, resulting in a total of 5 oligo probes per OR-contig. For design and arrangement of the Agilent microarrays, the eArray software (Agilent Technologies, Palo Alto, CA) was used. 

Labeling, hybridization and scanning of the microarray slides were done separately for queens, males and workers collected in the field (data set 1) and for large and tiny workers collected from the colony reared in the laboratory (data set 2). Each caste and subcaste was represented with four biological replicates in the microarrays except for workers of data set 1 with 3 biological replicates and 1 technical replicate of pooled RNA. The Low Input Quick Amp labeling kit (single color) from Agilent technologies (Agilent Technologies, Palo Alto, CA) was used for cDNA writing. For the generation of cDNA, 100 ng purified total RNA of workers, queens and males (data set 1), and 70 ng purified total RNA of large and tiny workers (data set 2) was used as template. Cy3 labeled cRNA was produced from the non-labeled cDNA using the Kreatech ULS Fluorescent labeling kit (Kreatech, Amsterdam, Netherlands). In order to purify the labeled cRNA, the RNeasy mini Kit (Qiagen, Hilden, Germany) was used. At last, the quality of the purified and labeled cRNA was tested by measuring the specific activity of the dye with a NanoDrop ND-1000, using the Microarray function. All replicates contained >3 µg cRNA with a specific activity of >6 pmol Cy3/µg cRNA. A total amount of 1,65 µg of labeled cRNA per chip was used for the hybridization procedure and chips were hybridized in a loop-design. All solutions used for hybridization, washing and drying were acquired from Agilent Technologies (Agilent Technologies, Palo Alto, CA). Hybridization took place at 65°C for 17 h. Microarray chips were washed in GE buffer, following the instructions of the one-color microarray-based gene expression analysis manual from Agilent Technologies (Agilent Technologies, Palo Alto, CA) and preserved with the stabilization and drying solution before scanning with the Agilent C Scanner (Agilent technologies, Palo Alto, CA). Intensity values for the individual oligos were obtain with Agilent Feature Extraction software and background intensity was automatically subtracted (Agilent technologies, Palo Alto, CA). 

The microarray data are available at Gene Expression Omnibus (http://www.ncbi.nlm.nih.gov/geo/) under the accession number GSE 48824 and were analyzed using the Bioconductor package in R [53,54]. Both data sets were normalized separately using quantile normalization, and the four biological replicates of the oligos designed on basis of the same contig were pooled. Microarray data of queens, males and workers (data set 1) were analyzed by calculating an analysis of variance (ANOVA) on the basis of single contigs. Subsequently, p-values were corrected for multiple testing using the Benjamini-Hochberg approach, and the Student-Newman-Keuls post-hoc test (SNK-test) was calculated. For the comparison of large and tiny workers (data set 2), a Student's t-test (two tailed, unpaired) instead of an ANOVA was calculated and p-values corrected for multiple testing using the Benjamini-Hochberg approach. A threshold value of 100 for absolute mean intensities was used to exclude weakly expressed genes from the analysis, and ratios of the intensity values were considered as biologically relevant when equal or exceeding 2-fold difference. 

In order to identify OR-genes that potentially code for pheromone receptors, we searched for overall high expression levels and differential expression in a biologically relevant range (2fold difference). As a measure for the expression level of a gene/gene fragment, we calculated the corresponding intensity value with respect to the variance of intensity values of all OR-gene and OR-gene fragment related oligo probes, using the following formula: EF = (LTI_cand_ - LTI_mean_) / SD

The expression factor (EF) is based on the difference of log-transformed intensities (LTI) of an OR-gene or OR-gene fragment related oligo probes (mean of biological replicates; LTI_cand_) and the mean of all OR-gene and OR-gene fragment related oligo probes (LTI_mean_), divided by the standard deviation of LTI_mean_ (SD).

## Results and Discussion

### Initial sequencing and transcriptome

Pooled cDNA from queens, males and workers of *A. vollenweideri* was used as a template for Illumina Solexa and Roche 454 FLX sequencing, generating 4,4 billion bases of information (Table 1). Reads were assembled resulting in 45.383 contigs larger than 150 bp. Initial automatic annotation using Blast2GO [43] identified putative homologs for 48% of the contigs, assigning Gene Ontology (GO, see [55]) terms to 45% of these hits. 

**Table 1:** Assembly output of the antennal transcriptome data.

contigs with length >150 bases	45383
N25	1212
N50	719
N75	424
GC-content	40,69%

**Figure 1:**
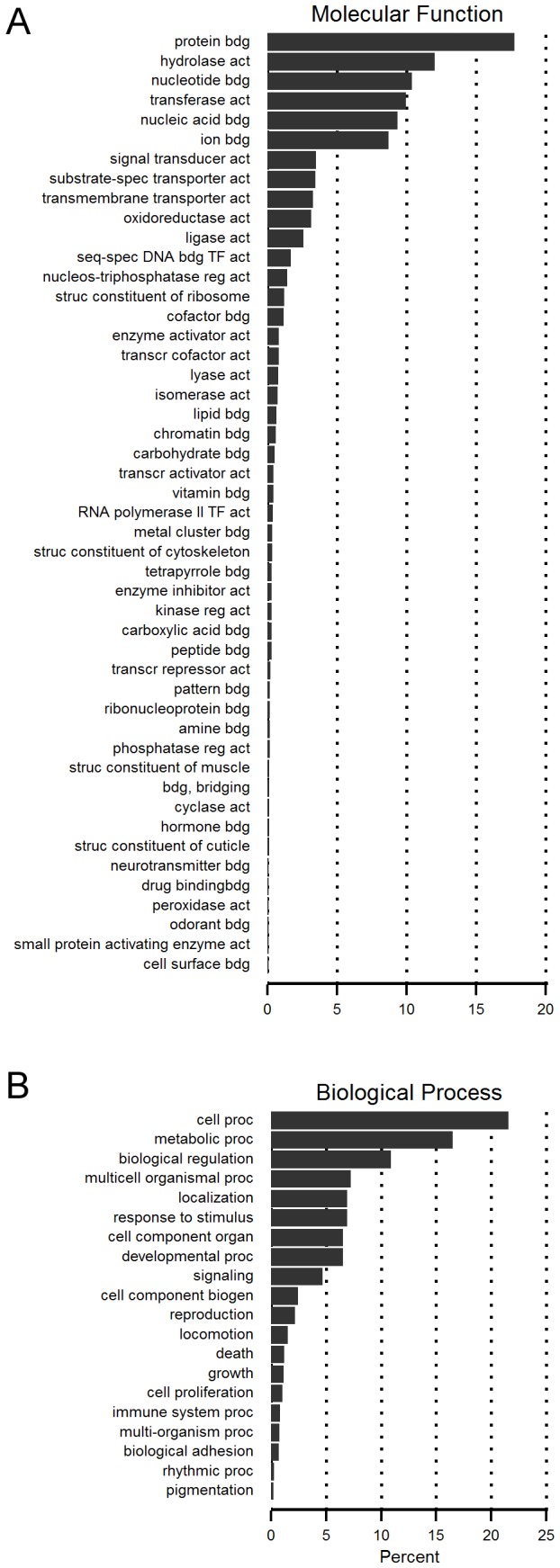
GO-term analysis in *A. vollenweideri*. Frequencies of GO-terms for antennal transcriptome sequences and their classification in Molecular Function (**A**) and Biological Process (**B**). GO-terms in **A** are presented as level 3 categorization, and GO-terms in **B** are presented as level 2 categorization. Note that a contig can be assigned to more than one category.

For further quality assessment of the transcriptome sequence data, we calculated the frequency of GO terms in molecular function (on level 3) and biological process (on level 2; Figure 1). In the general analysis of molecular function, 21.4% of the annotations are linked to enzymatic activity (terms: “hydrolase activity” and “transferase activity”). A further 22% of annotations are connected to terms like e.g. “nucleotide binding”, and “nucleic acid binding”. Regarding metabolism, “lipid binding” is assigned to 97 contigs and “carbohydrate binding” to 74. The GO analysis for biological processes reveals a large number of annotations belonging to “cellular process” (21.6%) and “metabolic process” (16.5%); 6.9% of all annotated contigs are assigned to “response to stimulus”. From the data we conclude that on an abstract level as described by GO annotation, the antennal markup of *A. vollenweideri* is comparable to similar transcriptome datasets in other insect species, for example the Lepidoptera *Manduca sexta* [44] and *Spodoptera litoralis* [56]. One difference is the larger number of contigs associated with the term “response to stimulus” in *A. vollenweideri*. The ants’ central nervous system already indicates an order of magnitude higher complexity of the peripheral olfactory sense, and this difference fits the idea of a dedication to higher sensory resolution on a molecular level. 

### Caste and subcaste-specific GO

**Figure 2:**
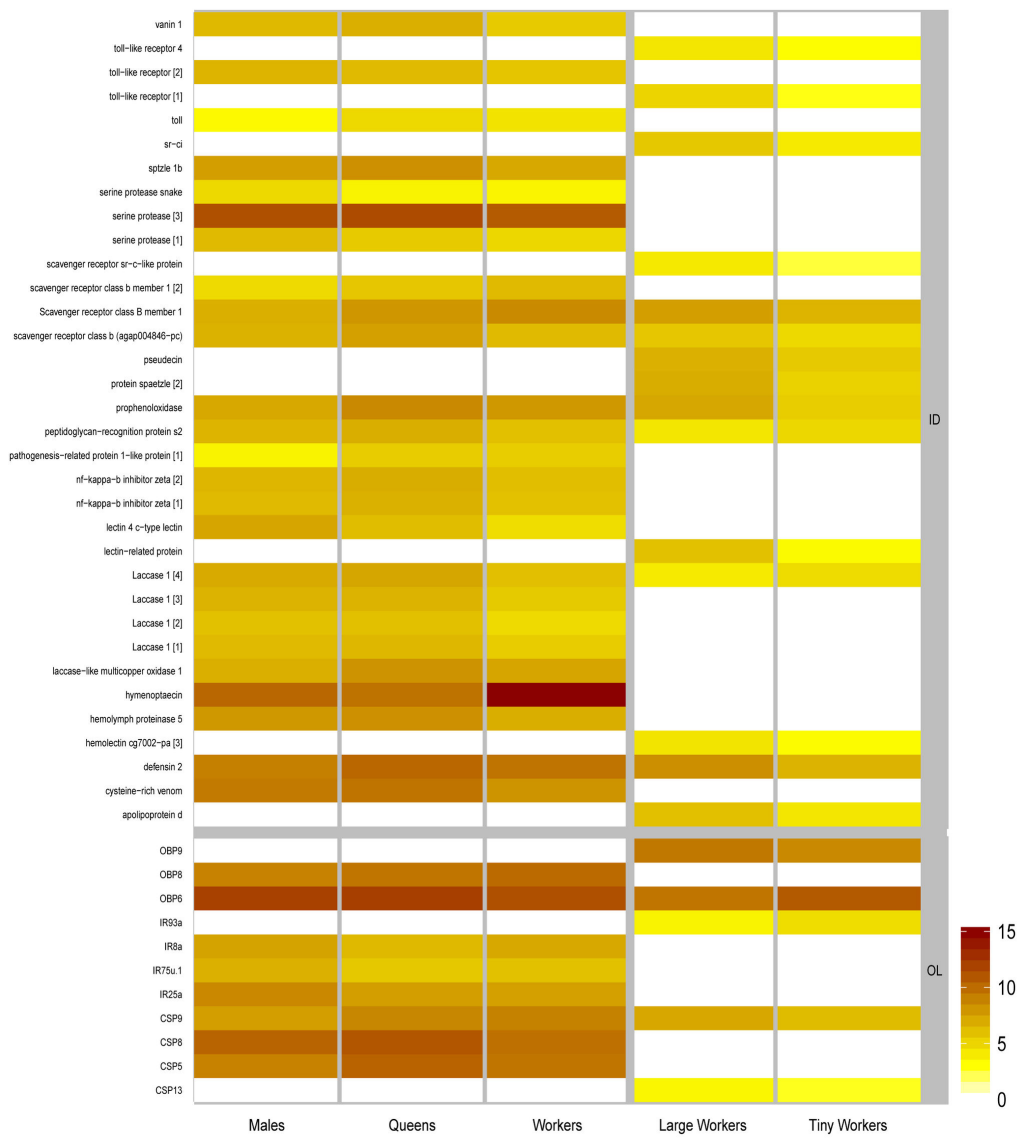
Heatmap of differentially expressed genes across castes and subcastes. Mean intensities of biological replicates with annotated gene function were log-transformed. As cutoff level for expression, contigs with mean intensity values of less than 100 were excluded from the analysis. Expression levels are considered as biologically relevant, when ratios across the comparison groups exceed a 2-fold difference. Queens, males and workers (left-panel) were tested separately from large and tiny workers (right-panel). White boxes indicate that the respective genes are either not expressed above cutoff level or were not expressed differentially at biologically relevant levels.

Caste-specific gene expression was reported previously in other species of Hymenoptera for representatives of various gene families involved in olfaction and immune defense [38,57-59]. In order to investigate similar caste and subcaste-specific effects in *A. vollenweideri*, we designed custom microarrays based on the antennal transcriptome data and examined antennal cDNA of different castes and subcastes for gene expression (see Table S1; Figure 2). Presence-absence calls from individual microarrays are used as basis for abstract caste and subcaste-specific GO-analysis. 

Numbers of contigs associated with the respective GO-terms for molecular function or biological process neither differ significantly between queens and males nor between large and tiny workers (chi-square test, independently for each term; all p-values > 0.05, Figure S1). Thus, specific terms are represented similarly in castes and subcastes. However, it needs to be noted that this does not exclude differences on the level of single genes, neither regarding presence/absence nor differential expression. 

### Immune response

Based on automatic annotation, we identified contigs associated with the GO-term “immune system process”. Those contigs were manually revised by further analysis using the tblastx algorithm in combination with the NR (non-redundant) database of NCBI and by alignments of gene family members which were then visually inspected for redundancy. Because immune genes are rather conserved across insect species, we were able to assign unigene status to all gene fragments belonging to the immune response gene families. Finally, we tested the gene expression in the different castes and subcastes. Expression levels for specific genes are depicted in a heat map (Figure 2).

Genes involved in the innate immune response display two distinct expression patterns between castes and subcastes: Most immune genes are highly expressed in queen antennae compared to males and workers, however, few genes are highly expressed in males. 

Several families of proteins recognize conserved structures on the surface of microbes. The haemolymph proteins of the peptidoglycan recognition protein family (PGRP) are members of one of those families and the respective genes are expressed at higher levels in queens, compared to workers and males. Lectin related proteins can bind polysaccharides of pathogens. The corresponding genes show higher expression levels in large workers compared to tiny workers (~15.3 fold difference). Both types of haemolymph proteins activate the prophenoloxidase (PPO) which plays a major role in innate immune response, catalyzing early events of melanization (for reviews see Schmid-Hempel [60] and Cerenius and Soderhall [61]). Melanin is deposited around damaged tissue to form a physical barrier preventing infection. Similar to its activators, the PPO gene is highly expressed in queens compared to males and workers. Haemolymph analysis or whole-body microarrays in other ant species (*Lasius niger* [58]:, *Cataglyphis velox* [62]: *Acromyrmex octospinosus* [63]:) reported similar effects, e.g. PPO levels positively correlating with worker body size. 

Interestingly, two very prominent effector genes of the AMP (antimicrobial peptide) class, *defensin* and *hymenoptaecin*, which were also identified in *A. mellifera* [64,65], exhibit different expression patterns, predominately in the sexuals. *Defensin* is highly expressed in queens compared to workers and males (1.7-3.2 fold difference). In contrast to *defensin*, *hymenoptaecin* is highly expressed in males (1.7-7.3 fold difference). Both effector genes are activated by different pathways, e.g. the toll-pathway activating *defensin* and the Imd-pathway activating *hymenoptaecin*. The expression pattern of *hymenoptaecin* correlates well with the expression of *relish*, a central transcription factor of the Imd-pathway. Genes involved in the toll-pathway have elevated levels either in queens or large workers. 

The antennae are constantly exposed to potentially pathogenic microorganisms and different pathogens trigger the activation of specific immune pathways. The high expression levels of effector genes indicate an adaptation to either upcoming or current exposure to different pathogens as an adjustment of the immune system. The different tasks and lifestyles are reflected in the immune gene expression of the different castes and subcastes but its functional significance needs to be investigated.

### Chemosensation

Sequence diversity of some chemosensory gene families is very high, thus precluding unigene identification based on gene fragments we obtained by transcriptome sequencing. To alleviate this problem, as well as to facilitate analysis of potential genus-specific expansions in *Atta*, we also analyzed the publicly available data on predicted transcripts of the published genome of *A. cephalotes* (acep_OGSv1.2_transcript.fa.gz, 17.05.2011) the same way as the transcriptome data of *A. vollenweideri*. Predicted protein alignments of chemosensory gene family members of *A. cephalotes* together with the respective sequences in *A. vollenweideri* allowed removal of potential duplicates. For our microarray analysis we included all gene fragments because two or more non-overlapping protein sequences might relate to a single gene or as well originate from two genes due to a very recent gene duplication. Expression levels of the corresponding genes in the different castes and subcastes are depicted in a heat map (Figure 2).

In our conservative phylogenetic analysis of chemosensory genes and gene fragments of leaf cutting ants (*A. cephalotes* and *A. vollenweideri*), we analyzed only overlapping and non redundant gene fragments (potential duplicates removed), whereas the remaining sequences, although identified as corresponding to members of the chemosensory gene families, were excluded (Table 2).

**Table 2:** Overview of chemosensory-related gene fragments and genes (used for phylogenetic analysis) in *A. vollenweideri*.

Gene-fragment		Gene
OBPs	9	8
CSPs	13	13 (12)
SNMPs	2	2
IRs	7	7 (5)
GRs	2	2
ORs	185	70

#### OBP-, CSP- and SNMP-genes

Genes coding for odorant binding proteins (OBPs) exhibit low homology across hymenopteran species [21,22,66]. In *A. vollenweideri* as well as in *A. cephalotes*, we found 9 OBP-genes or gene fragments that are clearly related to OBP function (fasta-file S1). The number of OBP-genes is highly variable across hymenopteran species, with *Atta* species having less than *N. vitripennis* (90), *A. mellifera* (21), and the red imported fire ant *S. invicta* (18) [21,66-68]. In *A. mellifera* only 16 out of 21 OBP-genes are expressed in the antenna with most of them exhibiting differential expression patterns [21]. We also found differential expression of a subset of OBP-genes in *A. vollenweideri* (*OBP6*, *OBP8*, and *OBP9*). All three have generally high expression levels in all castes. Across large and tiny workers, *OBP6* and *OBP9* are differentially expressed, whereas across sexuals and workers, *OBP6* and *OBP8* are differentially expressed (Figure 2). Since the *A. cephalotes* genome data contains only a low number of OBP-genes, the gene family likely underwent gene loss in the genus *Atta*. 

**Figure 3:**
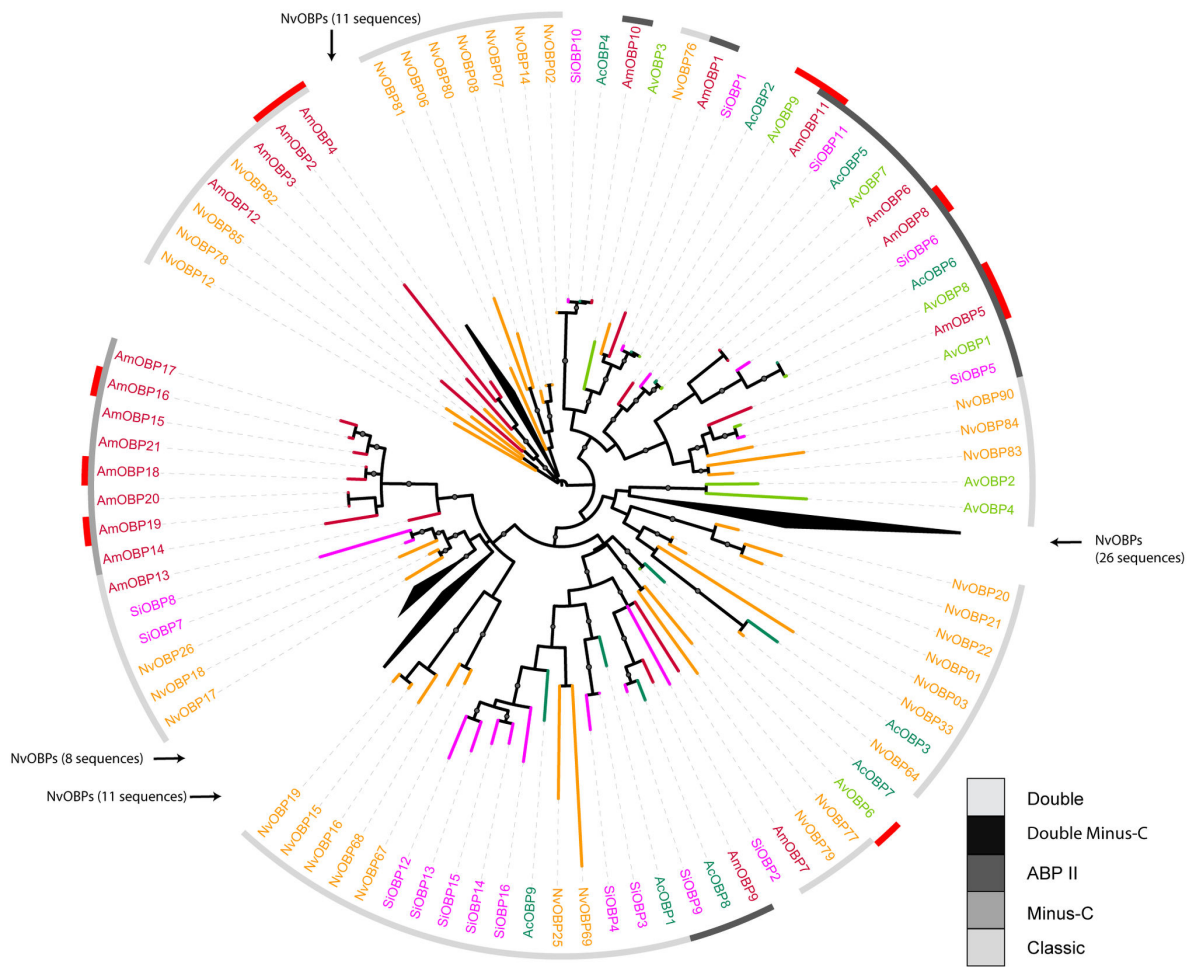
Phylogenetic relationship of the OPB protein sequences across different hymenopteran species. Protein sequences were aligned with MAFFT, and a neighbour-joining analysis in combination with a maximum-likelihood analysis was performed using FastTree. Local support values >0.8 are indicated by node labels. Color code for branches and labels: *A. vollenweideri* (light green), *A. cephalotes* (green), *S. invicta* (magenta), *A. mellifera* (red), *N. vitripennis* (orange). Code for the greyshade ring indicate OBP subfamilies and outermost ring indicates differentially expressed genes as red bars. Protein sequences are provided in a fasta-file (fasta-file S1).

Phylogenetic analysis of the OBP-gene family across diverse insect species revealed a classification into several subfamilies (based on sequence data: Classic, Minus-C, Plus-C, Dimer, Double, Double-Minus-C; and based on function: PBP/GOBP (abbr. from: Pheromone Binding Protein and General Odorant Binding Protein), ABP I (abbr. from: Antennal Binding Protein), ABP II, CRLBP (abbr. from: Chemical-sense-Related Lipophilic-ligand-Binding Protein), D7 (member of the Diptera salivary protein family), [22,66,69,70]. In *A. mellifera*, only three subfamilies (Classic, ABP II and Minus-C) have been described [21]. In our phylogenetic analysis, we could compare 8 OBP-genes of *A. vollenweideri* with other hymenopteran species. In contrast to *A. mellifera* and *N. vitripennis*, we did not find a genus-specific cluster for the two *Atta* species. Four OBP-genes (*OBP1*, *OBP7*, *OBP8* and *OBP9*) of *A. vollenweideri* have orthologs in *S. invicta* and for some of these we also find orthologs in *A. mellifera* and *N. vitripennis*. Three of those four OBP-genes belong to the ABP II subfamily (Figure 3, Figure S2). OBPs of the Classic subfamily have 6 conserved cysteins in their protein sequences, while in the Minus-C subfamily, a loss of one cystein at a specific position occurred, e.g. demonstrated in *A. mellifera* [21]. In both *Atta* species, OBPs either have the Classic 6 cysteins in their sequences (5 OBPs in each *Atta* species) or are too short to make a clear statement. Two OBP-genes of *A. cephalotes* (*OBP7* and *OBP9*) indicate a loss of one or two cysteins at different positions but whether they are members of the Minus-C subfamily is unclear due to poor sequence output of *A. cephalotes* genome data. Orthologs between *A. vollenweideri* and *A. mellifera* are only differentially expressed in one of the two species. Thus, we found no evolutionary conserved expression pattern, which suggests specific functions of different OBPs in different hymenopteran species. The low homology of OBP-genes within the Hymenoptera underlines that OBP-genes are highly divergent. The rapid rate of evolution together with the finding of specific OBP-gene expression in non-olfactory related tissues indicates that OBP-genes are often recruited for novel functions [21]. 

Like OBPs, chemosensory proteins (CSPs) are present in the sensilla lymph. Whether these proteins are involved in chemosensory processes is still under debate. We identified a total of 13 CSP-genes in *A. vollenweideri*, and in *A. cephalotes* we identified 14 CSP-genes (fasta-file S2). This number is larger than reported for other Hymenoptera, e.g. in *A. mellifera* a total of 6 CSP-genes was reported, with only 4 of them expressed in the antenna [71]. Microarray analysis revealed that 4 CSP-genes are differentially expressed across *A. vollenweideri* castes and subcastes. Three of the CSPs (*CSP5*, *CSP8* and *CSP9*) have high expression levels in queens (Figure 2). Regarding the worker subcastes, one CSP-gene (*CSP9*) which is highly expressed in workers, is also expressed at higher levels in large workers. All of the *A. cephalotes* CSP-genes and, because of the fragmentary nature of transcriptome data, only 12 CSP-genes were used in *A. vollenweideri* for phylogenetic analysis with other Hymenoptera. Interestingly, 8 CSP-genes of *A. vollenweideri* and *A. cephalotes* form a unique cluster, with no 1:1 orthologs in the non-ant Hymenoptera (Figure S3). The remaining four CSP-genes have orthologs in *A. mellifera*, and for three of them we find orthologs in *N. vitripennis.*

Sensory neuron membrane proteins (SNMPs) are membrane bound, belonging to the CD36 protein family and are presumably non-receptor proteins [72,73]. In *A. vollenweideri* as well as in *A. cephalotes* we identified two SNMP-genes, both belonging to the SNMP1-clade (*SNMP1a* and *SNMP1b*; Figure S4; fasta-file S3). All currently known SNMP-genes (*A. mellifera*, *N. vitripennis* and 13 dipteran species) belong either to the SNMP1- or the SNMP2-clade [74]. Expression of *SNMP1* is known to enhance pheromone detection in *D. melanogaster* [73]. In Lepidoptera, *SNMP1* is expressed in pheromone sensitive neurons, *SNMP2* in the surrounding support (sheath-) cells that express the corresponding pheromone binding protein genes [75]. Interestingly, none of the *Atta* SNMP-genes is differentially expressed across males and queens, suggesting that *SNMP1a* and *SNMP1b* have no prominent function in sex-pheromone communication.

#### IR-, GR- and OR-genes

Ionotropic receptors (IRs) are the most ancient chemosensory receptor type known to be involved in insect olfaction, including insects [24]. IRs are highly conserved in comparison to both, odorant receptors (ORs) and gustatory receptors (GRs) [23]. Functional IRs are heteromers consisting of a ligand-specific receptor and one of two IR co-receptors, coded by *IR25a* or *IR8a* [76]. We found seven IR-genes expressed in the antenna of *A. vollenweideri* and 18 IR-genes present in the genome of *A. cephalotes* (fasta-file S4). In other ant species, the number of identified IR-genes range from 23 in *H. saltator* to 32 in *L. humile*. All these numbers are considerably larger compared to IR-genes identified in *A. mellifera* and *N. vitripennis* (both 10) but not all of them are expressed in the antenna [24]. In comparison to *D. melanogaster* where 66 IR-genes were identified, only 17 are classified as antennal IR-genes [24]. In *A. vollenweideri* we could assign 6 out of 7 and in *A. cephalotes* 9 out of 18 of the IR-genes to the class of antennal IR-genes. All of the *A. cephalotes* IR-genes and, because of the fragmentary nature of transcriptome data, only 5 IR-genes were used in *A. vollenweideri* for phylogenetic analysis with other Hymenoptera and *Drosophila*. We included *Drosophila* because IR phylogeny is well described and the IR nomenclature was established in this group [24].

**Figure 4:**
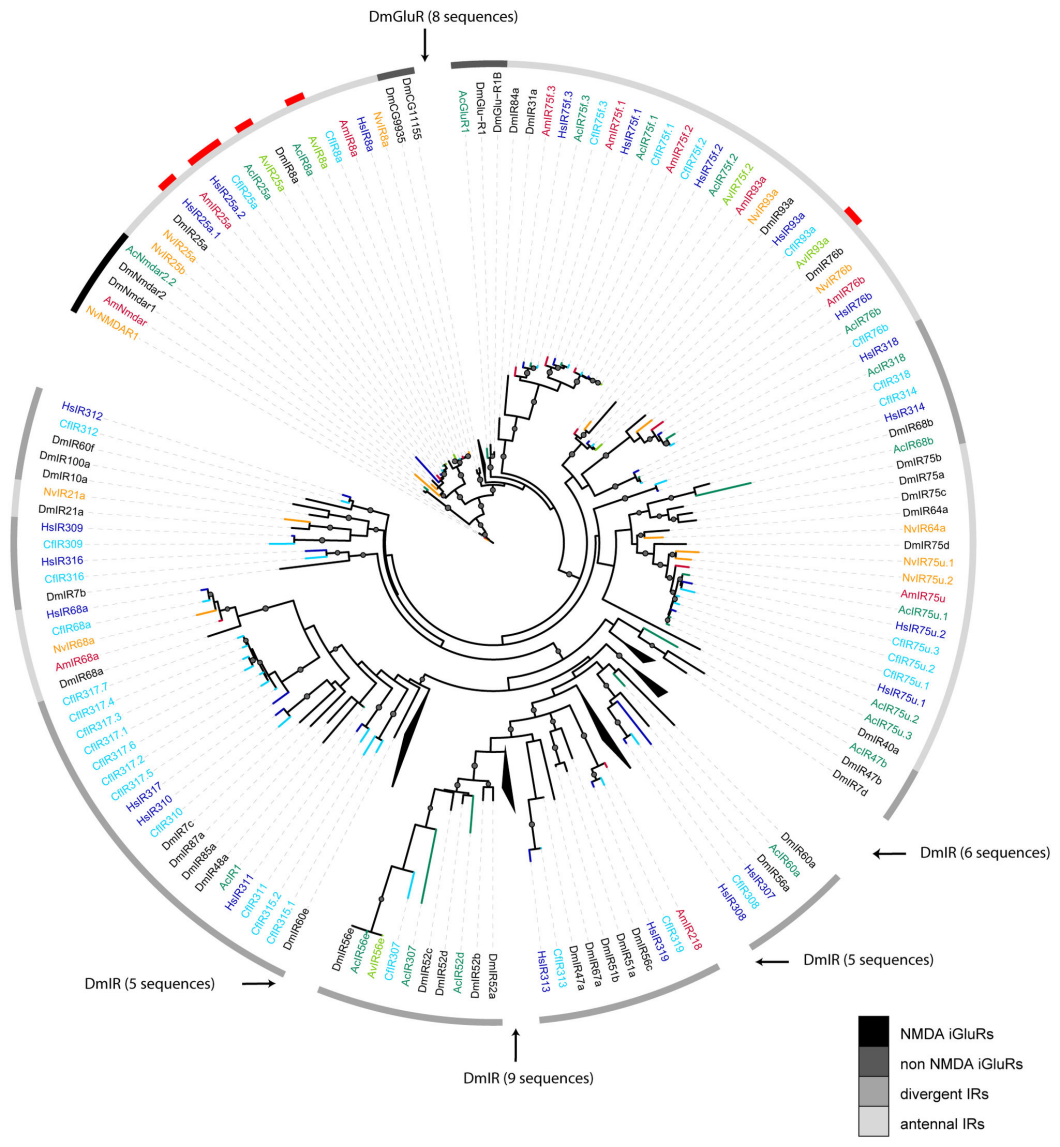
Phylogenetic relationship of the IR protein sequences across different hymenopteran species and *Drosophila melanogaster*. Protein sequences were aligned with MAFFT, and a neighbour-joining analysis in combination with a maximum-likelihood analysis was performed using FastTree. Local support values >0.8 are indicated by node labels. Color code: *A. vollenweideri* (light green), *A. cephalotes* (green), *A. mellifera* (red), *N. vitripennis* (orange), *D. melanogaster* (black), *H. saltator* (blue) and *C. floridanus* (light blue). Code for the greyshade ring indicate IR subfamilies and outermost ring indicates differentially expressed genes as red bars. Protein sequences are provided in a fasta-file (fasta-file S4).

In both *Atta* species we identified the coreceptor gene *IR25a* and *IR8a* (Figure 4 and Figure S5) and in *A. vollenweideri* the arthropod-specific *IR93a*. All of these highly conserved IR-genes (*IR25a*, *IR8a*, and *IR93a*) cluster together with the respective orthologs of other hymenopteran and dipteran species (Figure 4 and Figure S5). In some dipteran species, for example *D. melanogaster*, species-specific expansion are described for the divergent IR-genes [24]. Comparing different ant species, an expansion of divergent IR-genes was only described for *C. floridanus* [38] and could not be found in *A. cephalotes*. Interestingly, the antennal *IR75f* and *IR75u* subfamilies have three members in all ants species where there is only one ortholog in *D. melanogaster.*

In *A. vollenweideri*, a total of four IR-genes are differentially expressed between castes and subcastes (Figure 2). Both IR coreceptor genes (*IR25a* and *IR8a*) are highly expressed in males compared to queens and workers. Similar findings have been described for *Camponotus* and enriched expression of *IR8a* was found in *Harpegnathos* males [38]. In *D. melanogaster*, amine specific IRs form heteromers with the coreceptor IR25a [77]. Possibly, amines play an important but so far unknown role in the sex-pheromone communication of male ants. The high *IR75u.2* expression described for *H. saltator* males [38] and our finding that *IR75u.1* expression is enriched in *A. vollenweideri* males (rank_coreceptors_: 96%; rank_ligand-specific_ range: 70-84%), and that *IR93a* is highly expressed in tiny workers supports the idea that IRs provide specific functions in olfaction and potentially for communication in different castes of leaf-cutting ants. 

The second class of chemosensory receptors consist of GRs and ORs, originating form a distinct superfamily [28]. GRs are moderately conserved among different insect species and a subset is expressed in the antenna [78]. In *A. vollenweideri*, two GR-genes are expressed in the antenna, whereas we found 25 GR-genes in the genome of *A. cephalotes* (fasta-file S5), with presumably only a small subset expressed in the antenna. In *D. melanogaster* the function of some of the GRs was already assessed. For example CO_2_ detection is mediated by GRs (*DmelGR21a* and *DmelGR63a*, [79]). Orthologs of *GR21a* and *GR63a* were found in other insect species [80]. In our phylogenetic analysis, we specifically searched for *GR21a* and *GR63a* orthologs in both *Atta* species and our results confirm that these genes are not present in the Hymenoptera [29,30](Figure S6). Thus, we conclude that known CO_2_ detection in *Atta* is mediated by other, so far unidentified receptors [81,82]. 

Comparing our results with the 13 well-supported GR-subfamilies [38], we found that the two *A. vollenweideri* GR-genes are members of the GR1- and GR4-subfamily. The *A. mellifera* and *N. vitripennis* members of the GR1-subfamily were already described as sugar receptors, and it is likely that Hymenoptera only have two sugar receptor genes (*GR1* and *GR2*, [26]). In the genome data of *A. cephalotes*, we found homologs to both, *GR1* and *GR2*. The *A. cephalotes* GR-genes distribute across 9 of the 13 subfamilies and the F subfamily shows a small expansion (Figure S6). 

The main receptors in insect olfaction are the ORs. In *A. vollenweideri*, we identified 185 gene fragments related to ORs of which 70 could be identified as OR-genes. In comparison, we found 215 OR-genes in *A. cephalotes* (fasta-file S6). Functional ORs are heteromers consisting of a ligand-specific OR and ORCo. We identified the conserved ORCo-gene in *A. vollenweideri* but not in *A. cephalotes*, probably due to insufficient sequencing coverage of the *A. cephalotes* genome. Based on antennal lobe (AL) morphology with >400 glomeruli in large workers [13] and the very recent confirmation of the one glomerulus - one receptor gene hypothesis in several ant species [38], our analysis covered an estimated 46-50% of all OR-genes. One limiting factor of transcriptome sequencing is a potentially low expression level of genes, suggesting that the missing OR-genes are expressed at rather low levels in the antenna of *A. vollenweideri*. The fact that we could identify only a similarly low number of OR-genes in *A. cephalotes* indicates that *Atta* OR-genes are too diverse to be detected by automatic processes. 

Leaf-cutting ants exhibit caste- and subcaste-specific phenotypes regarding the total numbers of glomeruli in the AL with tiny workers and males having relatively small numbers of glomeruli in the AL (~380 vs 440 in large workers) [12,13]. Therefore, we expected differential expression of OR-genes and e.g. missing expression of some OR-genes in males and tiny workers. However, all of the identified OR-genes were represented in all castes and subcastes of *A. vollenweideri*, albeit at low levels for some. 

A recent phylogenetic analysis of hymenopteran OR-genes revealed 24 well-supported OR-subfamilies [38]. 

**Figure 5:**
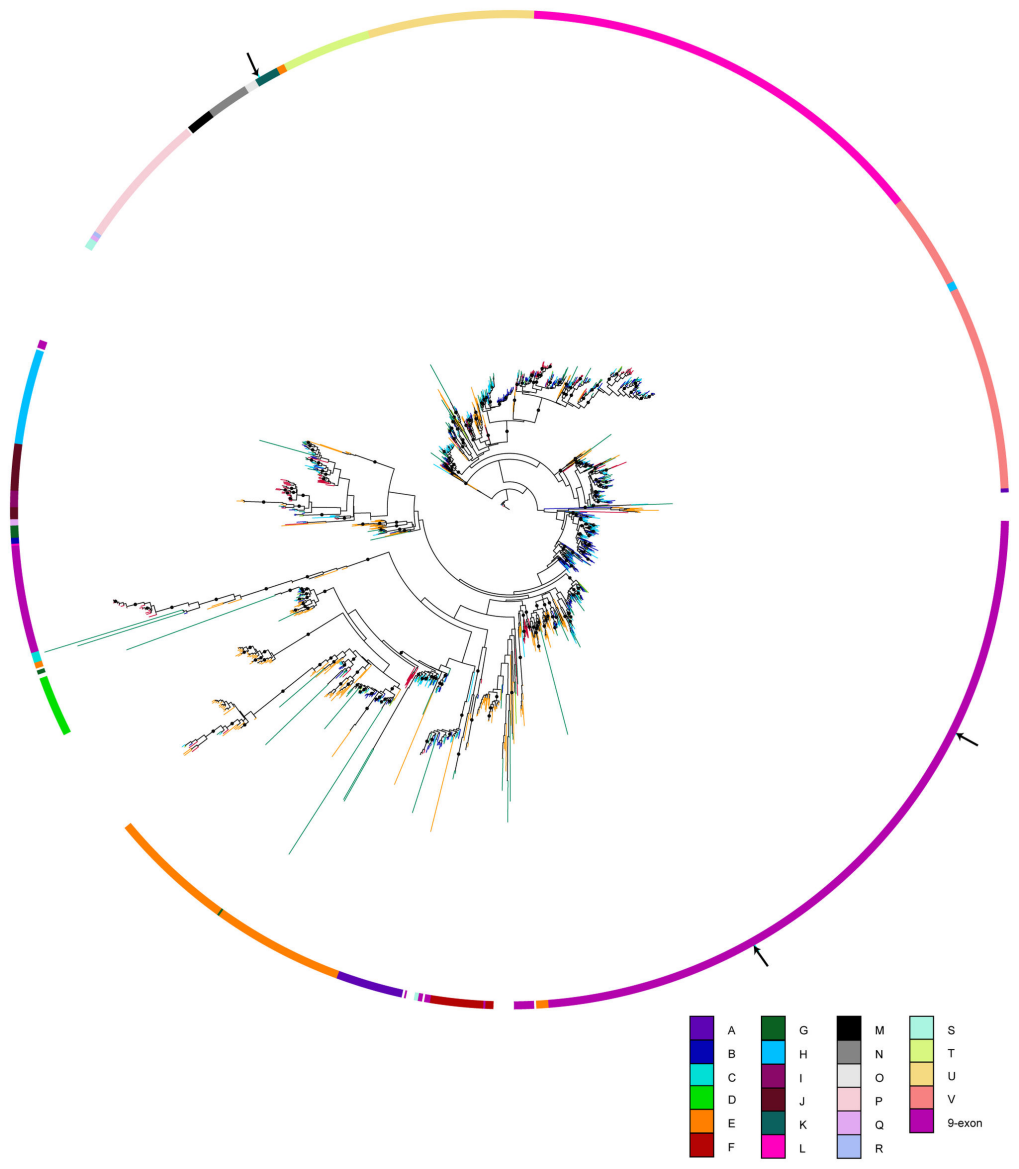
Phylogenetic relationship of the OR protein sequences across different hymenopteran species. Protein sequences were aligned with MAFFT, and a neighbour-joining analysis in combination with a maximum-likelihood analysis was performed using FastTree. Local support values >0.8 are indicated by node labels. Color code: *A. vollenweideri* (light green), *A. cephalotes* (green), *A. mellifera* (red), *N. vitripennis* (orange), *H. saltator* (blue) and *C. floridanus* (light blue). Code for the color ring indicate OR subfamilies and outermost ring indicates putative pheromone receptors as cyan bars. Protein sequences are provided in a fasta-file (fasta-file S6).

We compared all OR-genes found in *A. cephalotes* and, because of the fragmentary nature of transcriptome data used for OR-gene identification in *A. vollenweideri*, we could compare 70 of these with well-described OR-genes of ants (*C. floridanus* and *H. saltator*), and we included *A. mellifera* and *N. vitripennis* in order to strengthen phylogenetic relationships. The 215 OR-genes of *A. cephalotes* cluster in 18, and the 70 OR-genes of *A. vollenweideri* in 12 of the 24 subfamilies (Figure 5). The resulting phylogenetic tree shows intermittent subfamilies, as compared to [38], which is caused by our conservative approach of trimming the entire alignment to overlapping regions at the 3' ends of OR-genes. In most cases we found a pair of *A. vollenweideri* and *A. cephalotes* OR-genes clustering with respective OR-genes of *C. floridanus* and *H. saltator*. 

Species-specific expansions of OR-genes, e.g. one third of all OR-genes in the 9-exon subfamily, were reported for *C. floridanus* and *H. saltator* [38]. Our data do not support an *Atta-*specific expansions in the 9-exon subfamily, since in this subfamily we found only 16% (11) and 13% (28) of the OR-genes of *A. vollenweideri* and *A. cephalotes*, respectively. The transcriptome sequencing done for *A. vollenweideri* OR-genes might have induced a bias, due to missing OR-genes that are expressed at low levels, however, the genome data of *A. cephalotes* also did not support an *Atta*-specific expansion within the 9-exon subfamily. 

**Figure 6:**
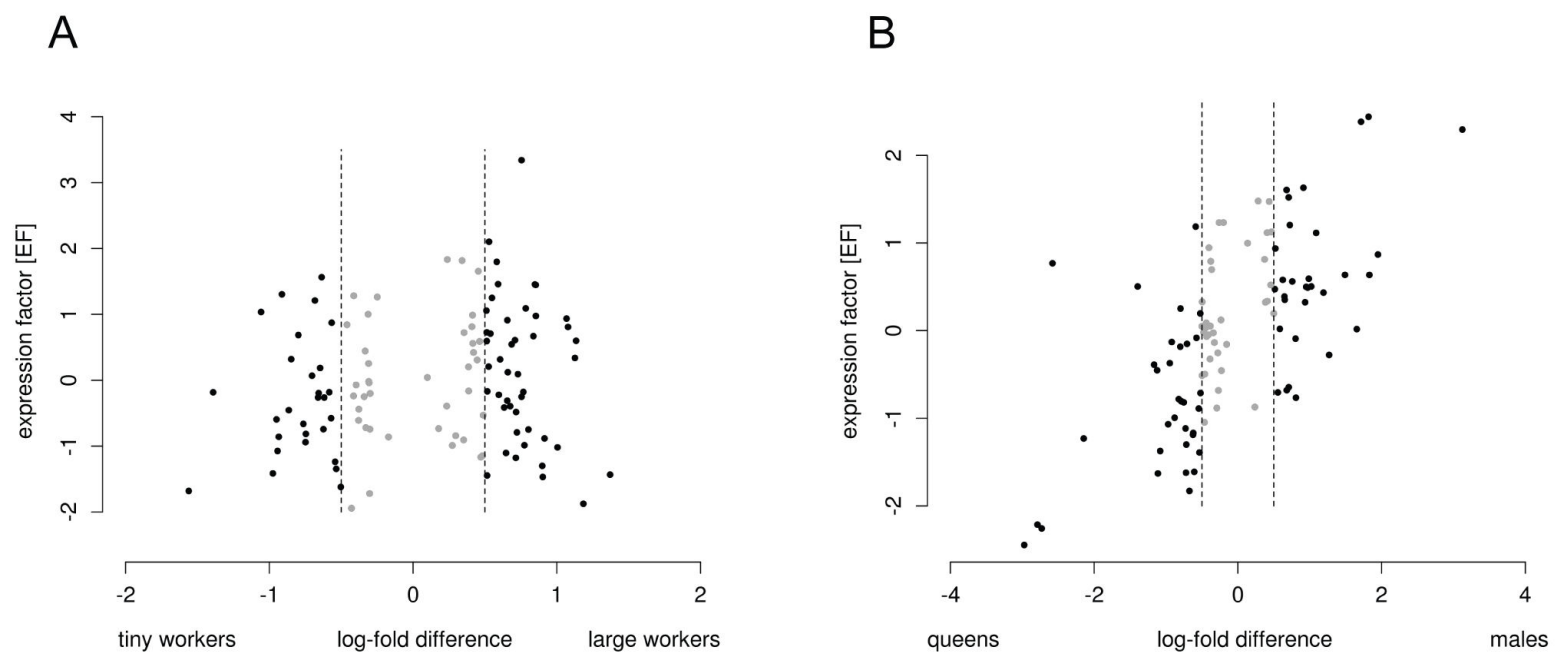
Comparison of OR gene expression between males versus queens and between large versus tiny workers. Relative OR gene expression is shown for large versus tiny workers (**A**) and for males versus queens (**B**). As a measure for the expression level of a gene, the corresponding intensity value was calculated with respect to the variance of intensity values of all OR genes using the following formula:.EF = (LTI_cand_ - LTI_mean_) / SD. The expression factor (EF) is based on the differences of log-transformed intensities (LTI) of the candidate gene (mean of biological replicates) and the mean of all OR genes, divided by the standard deviation (SD) of intensity values of all OR genes. In large workers, one OR gene is highly expressed, and a good candidate for the trail-pheromone receptor gene (**A**). In males, three OR genes are highly expressed and good candidates for sex-pheromone receptor genes (**B**). Log-fold differences between -0.5 and 0.5 are shown in grey and considered as biologically irrelevant differences.

Beyond the bare identification of expressed OR genes, we used differential expression analysis, related to AL-phenotypes in order to identify putative pheromone receptors. We expected high expression levels of single OR-genes in accordance with phenotypes containing a macroglomerulus (MG), namely in large workers and males. In large workers, one OR-gene (*AvOR131*) is highly expressed, with an expression factor (EF) of 3.4 and 2.1 fold-difference of expression in large versus tiny workers (Figure 6A, Table S2). All together: the presence of a single MG only in the AL of large workers [12,83], the representation of the releaser component of the trail-pheromone in the MG [13] and the high expression level of a single OR-gene in large workers provide strong correlative evidence that this OR-gene codes for a trail-pheromone receptor. 

In males, we identified three gene fragments with an EF of 2.3–2.4 and 5.5-22.7 fold-difference of expression compared to queens (Figure 6B, Table S2). For the phylogenetic analysis, one of the gene fragments had to be excluded because of our conservative trimming procedure. We are not able to ascertain whether it indeed codes for an independent receptor. With regard to the anatomy of the males' AL that contain three MGs [13], we at least could identify two OR-genes (*AvOR13* and *AvOR35*) that are promising candidates coding for sex-pheromone receptors. 

*AvOR131* (candidate for the trail-pheromone receptor) and *AvOR13* (candidate for one sex-pheromone receptor) belong to the 9-exon subfamily (Figure 5). The second OR-gene (*AvOR35*) that is highly expressed in males of *A. vollenweideri*, belongs to the K subfamily. In *C. floridanus*, male-enriched expression of OR-genes in the 9-exon subfamily have been described, and in *H. saltator* such OR-genes were found in subfamily E and subfamily L. In contrast to male-enriched expression of OR-genes in divers subfamilies in ants, OR-genes of moth that appear to be dedicated to sex-pheromone communication form a distinct phylogenetic subfamily [41,84]. Our results and the finding of male-enriched OR-genes in *H. saltator* in different subfamilies opens for speculations that in ants OR-genes involved in sex-pheromone detection have no common ancestor but rather were recruited from different subfamilies in the course of evolution [85]. 

Unfortunately, almost nothing is known about the chemical nature of sex-pheromones in ants. In sexuals of leaf-cutting ants, the content of several glands change in the course of nuptial flight and mating, but for none of the identified components distinct behavioral responses in males have been observed [5,86,87]. For other Myrmicine ants species, a single study reports 3-Methyl-1-(3-methylbutyl)-pyrrolidine as active component in sex-pheromone communication [88]. Queens produce this sex-pheromone component in their poison glands to attract males [89]. 

## Conclusion

Our study revealed pronounced differences in the expression of various genes and notably of olfactory related genes across the three castes and across worker subcastes of the leaf-cutting ant *A. vollenweideri*. These differences reflect and probably promote the complex social division of labor in the evolutionary derived genus *Atta*. We identified several genes in males and in large workers that may play important roles in pheromone detection. The results from our study are fundamental for functional characterization of the candidate genes and will provide the basis for identification of ligands (pheromone components). Beyond this aim, further comparative studies will lead to a better understanding on how the pheromone communication system in ants evolved.

## Supporting Information

Figure S1 **Caste and subcaste-specific GO-term analysis in *A. vollenweideri*.** Number of GO-terms for antennal transcriptome sequences and their classification in Molecular Function (A) and Biological Process (B) on level 3 are shown for males, queens, large and tiny workers. Note that a contig can be assigned to more than one category.
(TIF)

Figure S2 **Phylogenetic relationship of the OPB protein sequences across different hymenopteran species.** Protein sequences were aligned with MAFFT, and a neighbour-joining analysis in combination with a maximum-likelihood analysis was performed using FastTree. Local support values >0.8 are indicated by node labels. Color code for branches and labels: *A. vollenweideri* (light green), *A. cephalotes* (green), *S. invicta* (magenta), *A. mellifera* (red), *N. vitripennis* (orange). Code for the greyshade ring indicate OBP subfamilies and outermost ring indicates differentially expressed genes as red bars. Protein sequences are provided in a fasta-file (fasta-file S1).
(TIF)

Figure S3 **Phylogenetic relationship of the CSP protein sequences across different hymenopteran species.** Protein sequences were aligned with MAFFT, and a neighbour-joining analysis in combination with a maximum-likelihood analysis was performed using FastTree. Local support values >0.8 are indicated by node labels. Color code: *A. vollenweideri* (light green), *A. cephalotes* (green), *A. mellifera* (red), *N. vitripennis* (orange). Code for the greyshade ring indicate *Atta*-specific subgroup and outermost ring indicates differentially expressed genes as red bars. Protein sequences are provided in a fasta-file (fasta-file S2).
(TIF)

Figure S4 **Phylogenetic relationship of the SNMP protein sequences across different hymenopteran and dipteran species.** Protein sequences were aligned with MAFFT, and a neighbour-joining analysis in combination with a maximum-likelihood analysis was performed using FastTree. Local support values >0.8 are indicated by node labels. Color code: *A. vollenweideri* (light green), *A. cephalotes* (green), *A. mellifera* (red), *N. vitripennis* (orange). Code for the greyshade ring indicate SNMP subfamilies. Protein sequences are provided in a fasta-file (fasta-file S3).
(TIF)

Figure S5 **Phylogenetic relationship of the IR protein sequences across different hymenopteran species and *Drosophila melanogaster*.** Protein sequences were aligned with MAFFT, and a neighbour-joining analysis in combination with a maximum-likelihood analysis was performed using FastTree. Local support values >0.8 are indicated by node labels. Color code: *A. vollenweideri* (light green), *A. cephalotes* (green), *A. mellifera* (red), *N. vitripennis* (orange), *D. melanogaster* (black), *H. saltator* (blue) and *C. floridanus* (light blue). Code for the greyshade ring indicate IR subfamilies and outermost ring indicates differentially expressed genes as red bars. Protein sequences are provided in a fasta-file (fasta-file S4).
(TIF)

Figure S6 **Phylogenetic relationship of the GR protein sequences across different hymenopteran and dipteran species.** Protein sequences were aligned with MAFFT, and a neighbour-joining analysis in combination with a maximum-likelihood analysis was performed using FastTree. Local support values >0.8 are indicated by node labels. Color code: *A. vollenweideri* (light green), *A. cephalotes* (green), *A. mellifera* (red), *N. vitripennis* (orange), *D. melanogaster* (black), *H. saltator* (blue) and *C. floridanus* (light blue). Code for the color ring indicate GR subfamilies. Protein sequences are provided in a fasta-file (fasta-file S5).
(TIF)

Table S1 **Differentially expressed genes across castes and subcastes.**
(CSV)

Table S2 **Differentially expressed OR-genes and gene fragments between males versus queens and between large versus tiny workers.**
(CSV)

Fasta-File S1 **Protein sequences of OBP-genes and gene fragments of *A. vollenweideri* and *A. cepahlotes*.**
(FASTA)

Fasta-File S2 **Protein sequences of CSP-genes of *A. vollenweideri* and *A. cepahlotes*.**
(FASTA)

Fasta-File S3 **Protein sequences of SNMP-genes of *A. vollenweideri* and *A. cepahlotes*.**
(FASTA)

Fasta-File S4 **Protein sequences of IR-genes of *A. vollenweideri* and *A. cepahlotes*.**
(FASTA)

Fasta-File S5 **Protein sequences of GR-genes of *A. vollenweideri* and *A. cepahlotes*.**
(FASTA)

Fasta-File S6 **Protein sequences of OR-genes and gene fragments of *A. vollenweideri* and *A. cepahlotes*.**
(FASTA)

Dataset S1 **Alignment file of predicted protein sequences of OBP-genes.** For graphical representation of the phylogeny see Figure 3 and Figure S2.
(AFA)

Dataset S2 **Alignment file of predicted protein sequences of CSP-genes.** For graphical representation of the phylogeny see Figure S3.
(AFA)

Dataset S3 **Alignment file of predicted protein sequences of SNMP-genes.** For graphical representation of the phylogeny see Figure S4.
(AFA)

Dataset S4 **Alignment file of predicted protein sequences of IR-genes.** For graphical representation of the phylogeny see Figure 4 and Figure S5.
(AFA)

Dataset S5 **Alignment file of predicted protein sequences of GR-genes.** For graphical representation of the phylogeny see Figure S6.
(AFA)

Dataset S6 **Alignment file of predicted protein sequences of OR-genes.** For graphical representation of the phylogeny see Figure 5.
(AFA)

Dataset S7 **Phylogenetic relationship of the OPB protein sequences across different hymenopteran species shown in newick format.** For graphical representation of the same phylogeny see Figure 3 and Figure S2.
(AFA)

Dataset S8 **Phylogenetic relationship of the CSP protein sequences across different hymenopteran species shown in newick format.** For graphical representation of the same phylogeny see Figure S3.
(AFA)

Dataset S9 **Phylogenetic relationship of the SNMP protein sequences across different hymenopteran and dipteran species shown in newick format.** For graphical representation of the same phylogeny see Figure S4.
(AFA)

Dataset S10 **Phylogenetic relationship of the IR protein sequences across different hymenopteran species and *Drosophila melanogaster* shown in newick format.** For graphical representation of the same phylogeny see Figure 4 and Figure S5.
(AFA)

Dataset S11 **Phylogenetic relationship of the GR protein sequences across different hymenopteran and dipteran species shown in newick format.** For graphical representation of the same phylogeny see Figure S6.
(AFA)

Dataset S12 **Phylogenetic relationship of the OR protein sequences across different hymenopteran species shown in newick format.** For graphical representation of the same phylogeny see Figure 5.
(AFA)
