# Correction: Caste-Specific Expression Patterns of Immune Response and Chemosensory Related Genes in the Leaf-Cutting Ant, *Atta vollenweideri*

**DOI:** 10.1371/annotation/f55d34cc-7d25-442e-94cd-13338ca85b4d

**Published:** 2013-12-10

**Authors:** Sarah I. Koch, Katrin Groh, Heiko Vogel, Bill S. Hannson, Christoph J. Kleineidam, Ewald Grosse-Wilde

The name of the fourth author was spelled incorrectly. The correct name is: Bill S. Hansson. The correct Citation is: Koch SI, Groh K, Vogel H, Hansson BS, Kleineidam CJ, et al. (2013) Caste-Specific Expression Patterns of Immune Response and Chemosensory Related Genes in the Leaf-Cutting Ant, Atta vollenweideri. PLoS ONE 8(11): e81518. doi:10.1371/journal.pone.0081518. 

