# Supplementary figures and images for: Caste-Specific Expression Patterns of Immune Response and Chemosensory Related Genes in the Leaf-Cutting Ant, Atta vollenweideri
Source: PLoS One. 2013 Nov 15;8(11):e81518. doi: 10.1371/journal.pone.0081518 (PMC3829964; doi:10.1371/journal.pone.0081518)

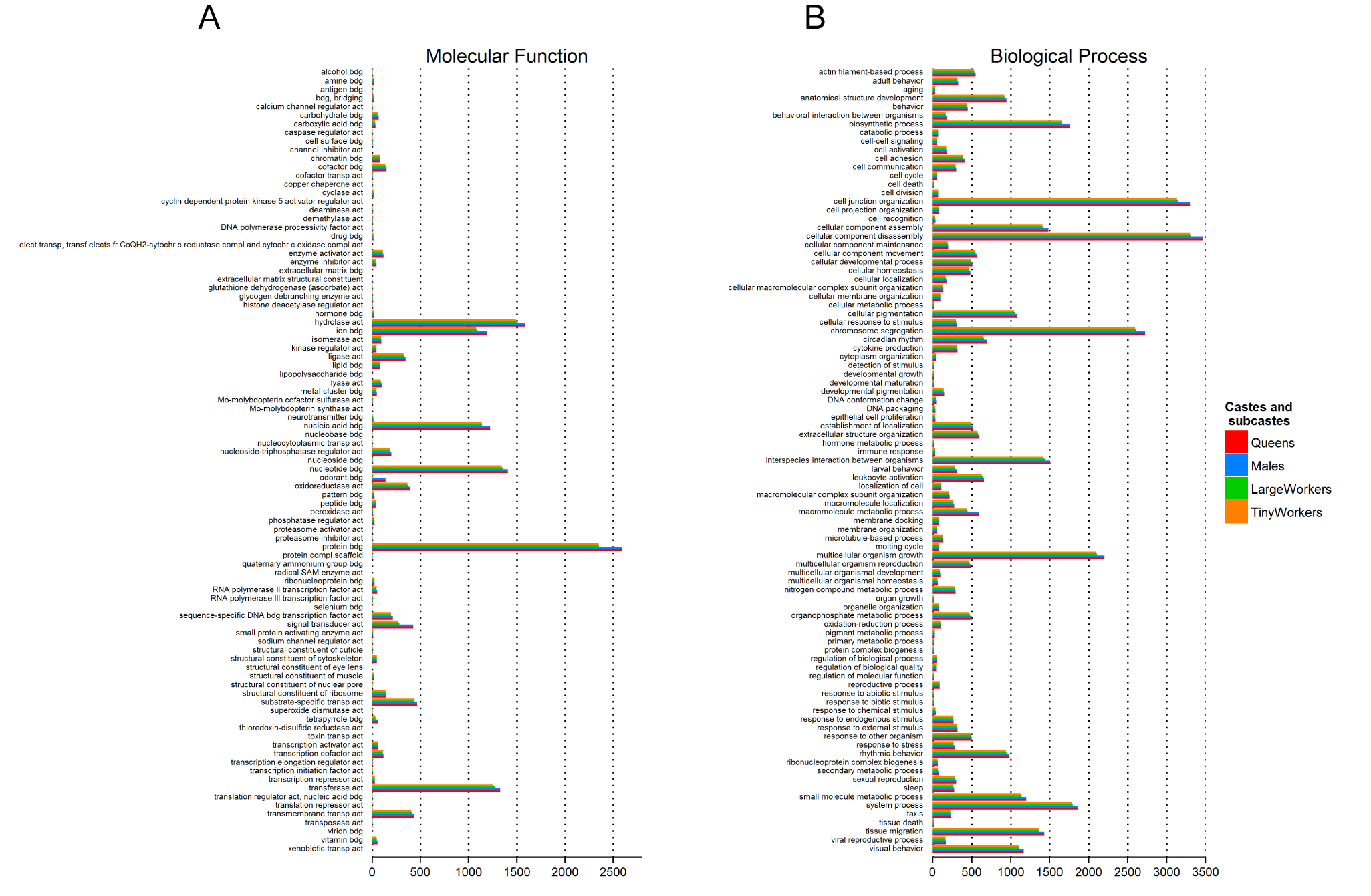

Supplement: Figure S1 — Caste and subcaste-specific GO-term analysis in A. vollenweideri. Number of GO-terms for antennal transcriptome sequences and their classification in Molecular Function (A) and Biological Process (B) on level 3 are shown for males, queens, large and tiny workers. Note that a contig can be assigned to more than one category. (TIF) [file pone.0081518.s001.tif]

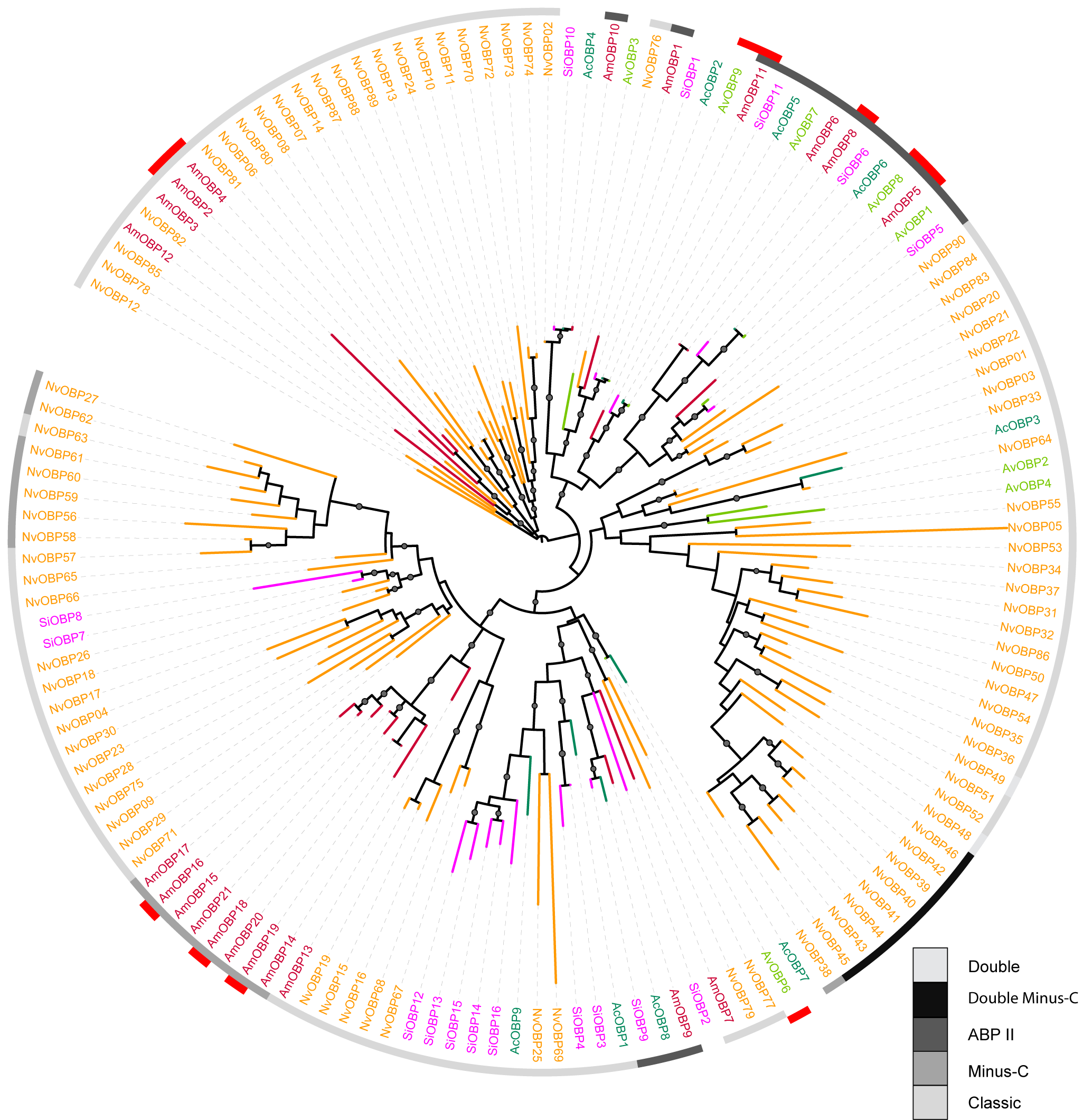

Supplement: Figure S2 — Phylogenetic relationship of the OPB protein sequences across different hymenopteran species. Protein sequences were aligned with MAFFT, and a neighbour-joining analysis in combination with a maximum-likelihood analysis was performed using FastTree. Local support values >0.8 are indicated by node labels. Color code for branches and labels: A. vollenweideri (light green), A. cephalotes (green), S. invicta (magenta), A. mellifera (red), N. vitripennis (orange). Code for the greyshade ring indicate OBP subfamilies and outermost ring indicates differentially expressed genes as red bars. Protein sequences are provided in a fasta-file (fasta-file S1). (TIF) [file pone.0081518.s002.tif]

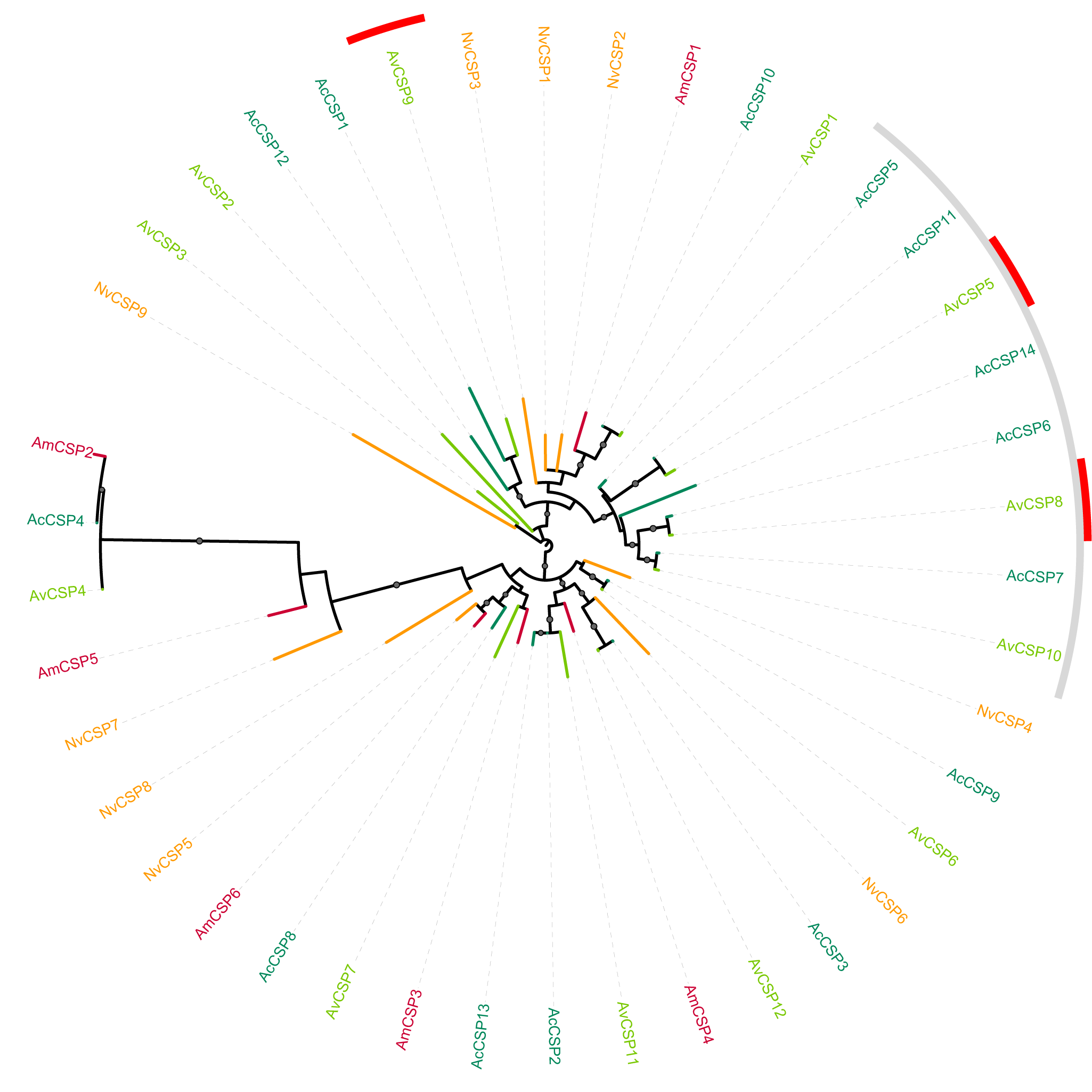

Supplement: Figure S3 — Phylogenetic relationship of the CSP protein sequences across different hymenopteran species. Protein sequences were aligned with MAFFT, and a neighbour-joining analysis in combination with a maximum-likelihood analysis was performed using FastTree. Local support values >0.8 are indicated by node labels. Color code: A. vollenweideri (light green), A. cephalotes (green), A. mellifera (red), N. vitripennis (orange). Code for the greyshade ring indicate Atta-specific subgroup and outermost ring indicates differentially expressed genes as red bars. Protein sequences are provided in a fasta-file (fasta-file S2). (TIF) [file pone.0081518.s003.tif]

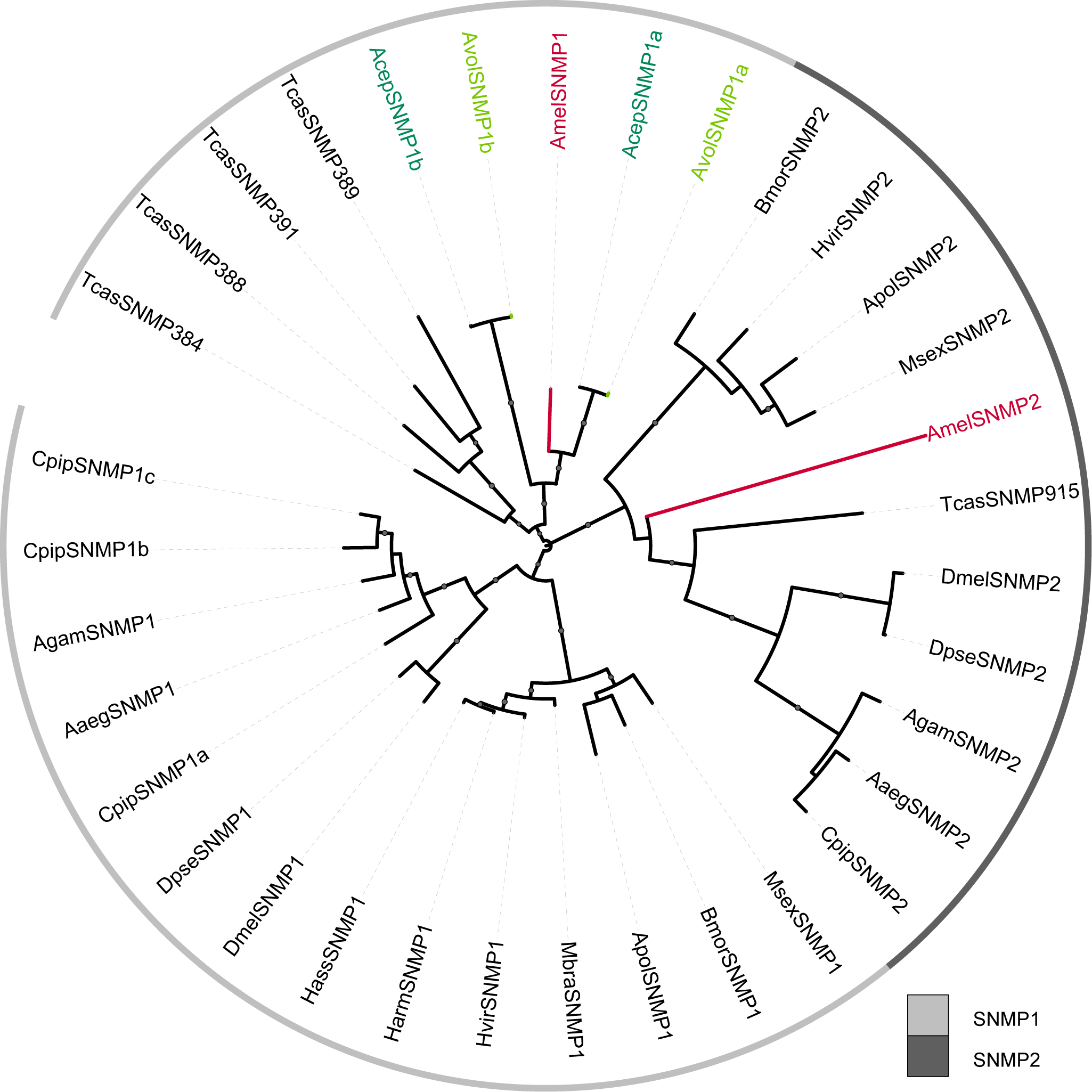

Supplement: Figure S4 — Phylogenetic relationship of the SNMP protein sequences across different hymenopteran and dipteran species. Protein sequences were aligned with MAFFT, and a neighbour-joining analysis in combination with a maximum-likelihood analysis was performed using FastTree. Local support values >0.8 are indicated by node labels. Color code: A. vollenweideri (light green), A. cephalotes (green), A. mellifera (red), N. vitripennis (orange). Code for the greyshade ring indicate SNMP subfamilies. Protein sequences are provided in a fasta-file (fasta-file S3). (TIF) [file pone.0081518.s004.tif]

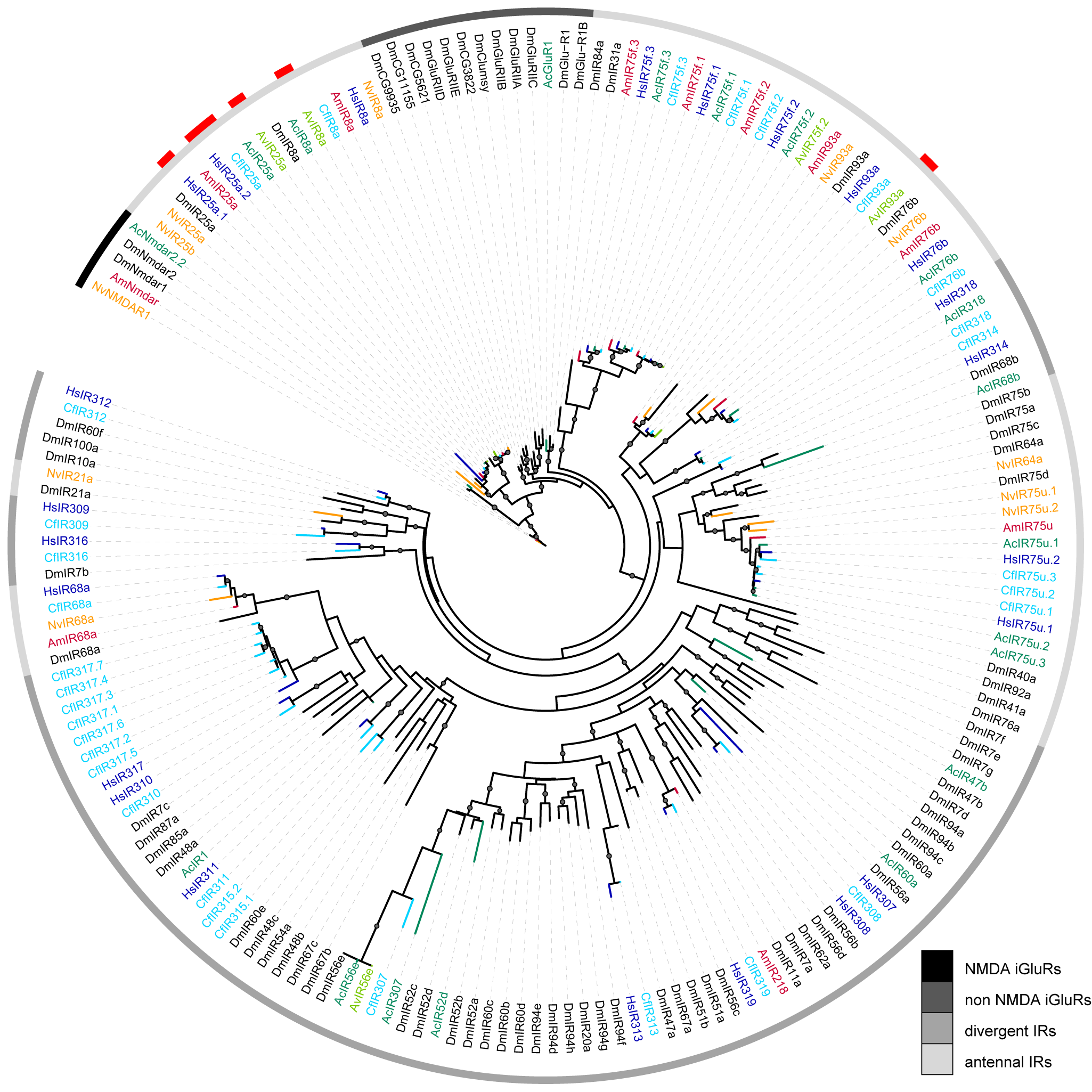

Supplement: Figure S5 — Phylogenetic relationship of the IR protein sequences across different hymenopteran species and Drosophila melanogaster. Protein sequences were aligned with MAFFT, and a neighbour-joining analysis in combination with a maximum-likelihood analysis was performed using FastTree. Local support values >0.8 are indicated by node labels. Color code: A. vollenweideri (light green), A. cephalotes (green), A. mellifera (red), N. vitripennis (orange), D. melanogaster (black), H. saltator (blue) and C. floridanus (light blue). Code for the greyshade ring indicate IR subfamilies and outermost ring indicates differentially expressed genes as red bars. Protein sequences are provided in a fasta-file (fasta-file S4). (TIF) [file pone.0081518.s005.tif]

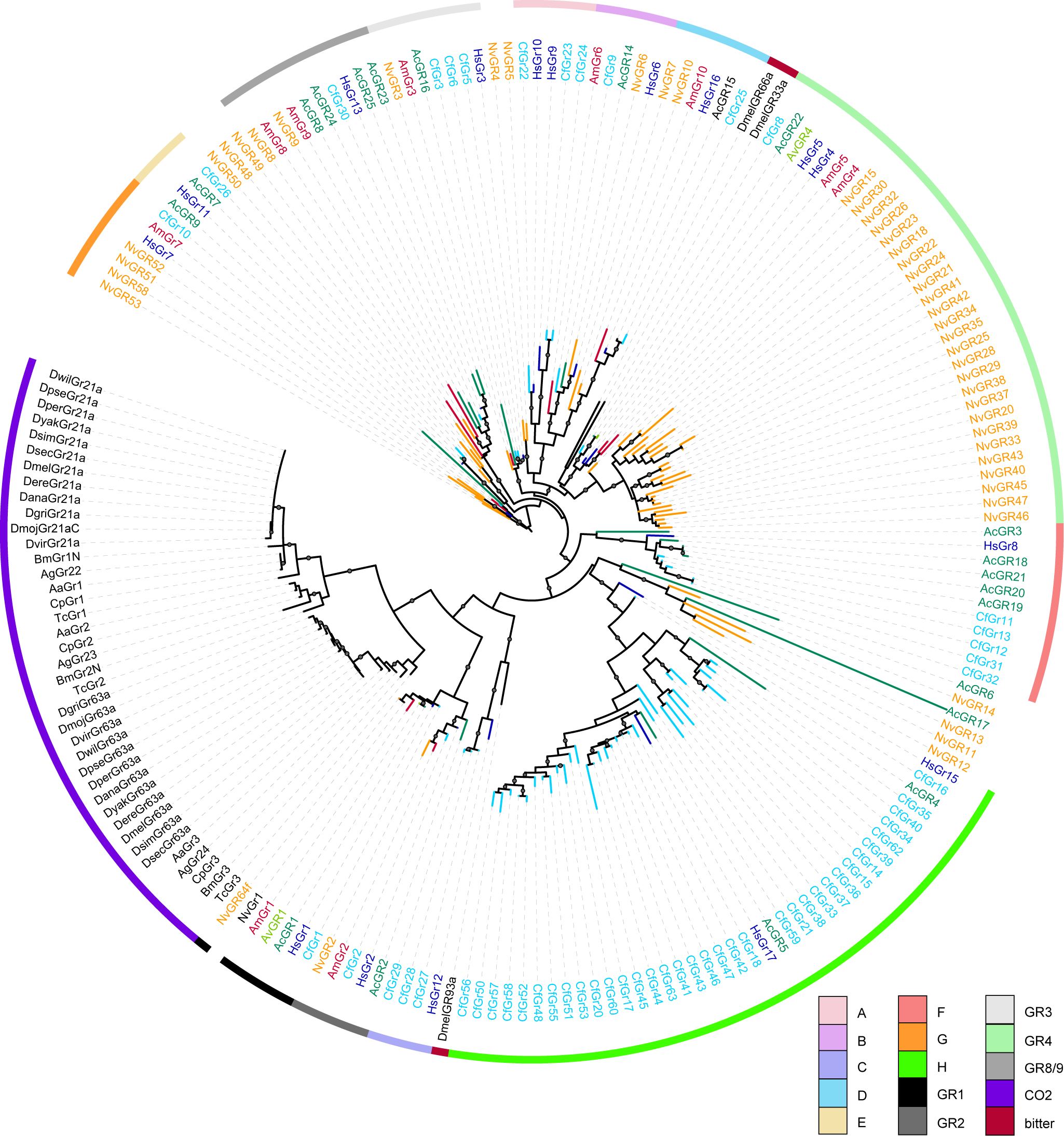

Supplement: Figure S6 — Phylogenetic relationship of the GR protein sequences across different hymenopteran and dipteran species. Protein sequences were aligned with MAFFT, and a neighbour-joining analysis in combination with a maximum-likelihood analysis was performed using FastTree. Local support values >0.8 are indicated by node labels. Color code: A. vollenweideri (light green), A. cephalotes (green), A. mellifera (red), N. vitripennis (orange), D. melanogaster (black), H. saltator (blue) and C. floridanus (light blue). Code for the color ring indicate GR subfamilies. Protein sequences are provided in a fasta-file (fasta-file S5). (TIF) [file pone.0081518.s006.tif]
